# Chronic Disease in Pediatric Population—A Narrative Review of Psychosocial Dimensions and Strategies for Management

**DOI:** 10.3390/children12080967

**Published:** 2025-07-23

**Authors:** Francesca Mastorci, Maria Francesca Lodovica Lazzeri, Lamia Ait-Ali, Pierluigi Festa, Alessandro Pingitore

**Affiliations:** 1 Clinical Physiology Institute, Consiglio Nazionale delle Ricerche Area della Ricerca di Pisa (CNR), 56124 Pisa, Italy; mariafrancescalodovicalazzeri@cnr.it (M.F.L.L.); lamia.ait-ali@cnr.it (L.A.-A.); alessandro.pingitore@cnr.it (A.P.); 2 Fondazione Gabriele Monasterio, 54100 Massa, Italy; gigifesta@ftgm.it

**Keywords:** children, chronic disease, quality of life, health, non-communicable diseases, parents, self-care

## Abstract

Children living with chronic diseases represent a great challenge for the health care system, their families, and communities. These young patients face continuous medical needs that affect not only their health but also their daily routines, emotional well-being, and family dynamics. In response, clinical practice is increasingly integrating psychosocial indicators alongside traditional medical parameters. Consequently, there is a growing consensus that the evaluation of pediatric chronic diseases should address not only clinical dimensions but also the disease’s impact on socialization, emotional health, and daily functioning. This narrative review explores the role of psychosocial variables in the management of pediatric chronic illnesses, including the experiences of parents and siblings, with a focus on effective strategies to improve everyday life. The integration of quality of life and well-being within a multidimensional care model could be instrumental in both symptom management and psychosocial support. Recognizing that children with chronic conditions are at increased risk for long-term adverse outcomes, it is critical to develop interventions that go beyond clinical care, encompassing education, coping reinforcement, and family-centered approaches.

## 1. Introduction

The emphasis of pediatric medicine from diagnosis to prevention and control of chronic diseases has been changed by advances in medical care. Moreover, the prevalence of pediatric chronic conditions increased, and the concern of health care providers and the health care system moved from mortality to lifetime morbidity and consequent correct emotional and behavioral development [1].

According to the Centers for Disease Control and Prevention (2024), chronic diseases are defined “as situations that last more than one year or more and require ongoing medical attention or limit activities of daily living or both”. The American Academy of Pediatrics states that chronic refers to a health condition that continues anywhere from three months to a lifetime, while for the WHO definition, “chronic diseases tend to be of long duration and are the results of combination of genetic, physiological, environmental and behaviors factors” [2,3]. Despite an increase in the prevalence of long-term conditions among children, there has been a relative dearth of focus on the impact of chronic disease within these younger age groups in the traditional clinical practice [4]. Although a cure is not possible, chronic diseases include a limitation of physical and social activities and require daily self-management throughout life [5].

In particular, chronic disease in childhood or adulthood is different and, consequently, disease management must be different between these groups [6]. Patients with chronic diseases have to adapt and develop a balance between the demands of life and those of the disease [7]. It has recently been shown that children with chronic diseases are about twice as likely during adolescence (10–13 years) to experience a mental health disorder as healthy peers [8]. Children and young people with chronic illnesses have a range of medical needs that can alter their daily routines and activities, and their families must develop a new lifestyle to cope with the daily management of the disease [9]. This lifestyle change can cause significant and persistent stress for children and their parents, with lasting emotional and social consequences that impact daily life and functioning [10,11].

Moreover, the scientific results on the adaptation of children with chronic disease show that these children experience not only lower physical functioning but also reduced emotional, social, and academic functioning compared to healthy children [12]. The findings, in turn, hurt health-related quality of life (HRQoL) [13,14]. Therefore, there has been a call for new outcome measures that reflect a more holistic approach to the management of these young patients, introducing the concept of health-related quality of life. This highlights the mind–body relationship and the link between physical and psychological health. Taken as a whole, there is growing interest in the idea that chronic disease assessments should be focused not only on clinical determinants but also centered on the impact that the disease may have on socialization processes, emotional health, and general limitations in ordinary activities. Furthermore, the biopsychosocial model, first introduced by Engel (1977), offers a valuable framework for understanding the complexity of chronic disease [15]. This model highlights the interplay between biological, psychological, and social factors, underscoring the need for an integrated and patient-centered approach to care [15].

The goal of this review article is to highlight the role and possible effects of subjective psychosocial variables in pediatric chronic diseases, emphasizing the strategies useful to manage these diseases according to a holistic and systemic approach, taking into account that neglecting non-clinical factors may lead to disease exacerbation.

This paper starts by presenting the burden of non-communicable diseases in pediatric populations. Next, psychological effects on quality of life are briefly discussed. The following section summarizes the daily functioning of living with a chronic condition. Lastly, a comprehensive model for health management and limitations is highlighted in the future perspectives section.

### Methods

We conducted a narrative review of the existing literature using PubMed and Scopus. The narrative review method was used to enable a thematic analysis of the psychological effects of chronic diseases in children. The articles were identified by conducting an abstract/title search with the following terms: chronic diseases AND psychological OR psychosocial AND children OR pediatric population.

Articles presenting on psychological effects, social processes, emotions, public health, education, and caregivers were included. An additional targeted search was conducted using Google Scholar to gather additional information. Snowballing of references was also used to identify relevant articles from the articles that came out of the search.

Priority topics were selected based on their frequency and emphasis across previous systematic reviews and meta-analyses focusing on pediatric populations with chronic illnesses, as well as their relevance to clinical practice and modifiability”.

## 2. Burden of Non-Communicable Diseases Among Children

Thanks to advances in the diagnosis and management of pediatric diseases, children can live longer and reach adulthood. According to their impact on daily life, pediatric chronic diseases are classified as mild, when limitations in daily activity are absent, moderate with limitations in some activities, and severe with great impact on physical status [16].

It is now well established that the number of children suffering from chronic diseases is increasing in the world’s population. It is estimated that approximately 10–18% of young people are affected by a chronic disease [17]. At first view, these data may seem discouraging, as they indicate an increase in the prevalence of diseases such as diabetes mellitus, chronic asthma, or congenital heart disease in childhood. However, it is worth considering that this increase is due in part to advances in the medical–scientific field, which have enabled us to make diagnoses early, thus increasing the life expectancy of these young patients. Pathologies that were once considered fatal can now be treated and managed more accurately with targeted therapeutic interventions. Usually, chronic diseases start as an organ disease and become systemic in their progress. In this evolutionary process, different disease conditions share common pathophysiological mechanisms, both in adults and in pediatric patients, such as the activation of hormonal, inflammatory, and nervous systems (autonomic nervous systems) that initially are protective but become potentially harmful when continuously activated [18].

In general, although there is no unambiguous definition of a chronic disease, the emphasis is on the persistence of the disease over time; these are conditions that cannot be completely cured with the current state of medical knowledge. Therefore, they require a difficult adaptation process, an active and conscious role on the part of the subject, in our case, of children and family [19]. Some chronic diseases are characterized by a progressive and inevitable psychophysical decline, while others, thanks to adequate and continuous pharmacological treatment, can halt their course. In most cases, however, these are conditions characterized by alternating periods of remission and relapses, in which the prognosis is often uncertain and characterized by numerous hospitalizations and frequent access to health services. Therefore, the adaptation of children with chronic disease concerns not only physical functioning but also emotional, social, and school functioning [19].

Pediatric diseases are strictly interconnected with the degree of development and economic status of the country. Accordingly, in low- and middle-developed countries, the primary causes of diseases and mortality for children under 5 years of age are pneumonia, diarrhea, measles, malaria, and malnutrition; on the contrary, in developed countries, children’s health is essentially correlated with non-communicable diseases (NCDs) [20]. NCDs are a set of chronic diseases that are not transmitted from person to person, have a long duration with slow progression, and are rarely completely curable, including cardiovascular diseases, cancers, respiratory diseases, and diabetes. In recent years, in the pediatric population, we have observed an increase in the prevalence of chronic disease, both isolated and in comorbidity [21].

This growth is due to different factors, such as the prevalence of a wide range of other health problems, such as hepatic, renal, and gastrointestinal diseases; endocrine, hematological, and neurological disorders; genetic conditions; mental disorders; and other disabilities, and advances in medical care that have determined an increase in survival [22,23]. Epidemiological studies show that 1 in 4 children have a chronic disease, with prevalence estimates ranging from 10% to 30% [24]. Of the chronic conditions, respiratory disorders and cardiovascular diseases (CVDs) are the most frequent in the pediatric population, with a global increasing trend in developed countries [25,26]. The most recent 2019 epidemiological data show that more than 16.8 million children are affected by CVDs [27]. In the pediatric population, CVDs include congenital heart disease, rheumatic heart disease, and stroke, and about 90% live in areas where adequate medical care is absent or insufficient. Among chronic respiratory diseases (CRDs), asthma is the most common condition affecting children and is estimated to afflict 14% of all children globally. This chronic condition in childhood is related to CRDs in adulthood. In fact, early respiratory infection or environmental exposures may lead to CRDs in adulthood. Worldwide, the frequency of diabetes is increasing among NCDs. Since 1990, the worldwide prevalence of children with diabetes, especially type 2, increased from 2.7 million to 4.5 million in 2019. Alongside these more traditional conditions, recent years have seen an increase in mental disorders, including anxiety, attention deficit, autism spectrum, behavior, mood, and eating disorders [28]. In 2019, over 227 million children showed mental disorders, with long-term consequences in terms of years of life lost due to disease (YLD). Independently of one condition or the other, a set of risk factors, such as pollution, obesity, high blood pressure, and poor sanitation, can increase the likelihood of developing a disease; thus, understanding risk factors is pivotal to defining preventive programs and strategies.

## 3. Psychological Effects on Quality of Life in Children with a Chronic Disease

According to the WHO, health is considered “a state of complete physical, mental and social well-being.” The concept of quality of life is even more complex. Quality of life is a multidimensional construct influenced by several factors; it is how individuals perceive their lives about the environment, culture, and social contexts in which they live, and how their judgements influence their life goals and expectations; in other words, unlike the more objective assessment of health, quality of life is more subjective [29]. Given its complexity, it is influenced by physical health, psychological state, level of independence, social relations, and environmental characteristics. Recently, quality of life has been considered a determining factor in the follow-up and management of chronic diseases in children, even on the same level as the traditional clinic, and is closely correlated with treatment success. In general, most clinical studies have shown significant differences between the responses of children and their parents concerning quality of life, with pediatric patients tending to report higher quality of life scores than their parents [30]. Recent data suggest that up to 30% of children with chronic illnesses exhibit clinically significant emotional or behavioral symptoms, with variation depending on age, sex, and condition severity [4]. These estimates also vary according to the informant: studies consistently show that parents tend to report more severe psychosocial issues than children themselves or health professionals, underlining the need for multi-informant assessments in both research and clinical settings [30]. Many studies underline how living with children suffering from a chronic disease profoundly affects the life habits of the whole family, both parents and siblings, increasing stress levels considerably [31]. Parental distress has been reported in 30 to 80% of parents with consequences on parental mental health, parenting, parent–child relationships, and parental quality of life, but does not appear to be related to the severity of the disease [32]. From a psychological point of view, parents, and especially mothers, are at risk of distress, anxiety, depression, somatization, hopelessness, and post-traumatic stress symptoms, which in turn may influence mothers’ responsiveness; physically, they are tired, with scarce energy, and mentally frustrated. Therefore, considering also indirect effects on the household and given the ever-increasing number of children with chronic diseases, it is necessary to take into account aspects that are secondary to the disease, but profoundly influenced by it, such as the psychosocial and emotional dimensions. Indeed, despite significant improvements in the survival rates of chronically ill children, they remain a vulnerable group. These maladaptive behaviors may exacerbate the risk of developing further adverse effects such as depression, self-harm, body esteem issues, altered physical functioning, obesity, and poor quality of life [33]. Understanding these aspects is important because it helps to identify the objectives of the prevention and intervention to implement. In this context, three categories of functioning should be considered: social functioning, which concerns the child’s ability to perform in social contexts, such as making friends; school functioning, regarding those behaviors aimed at good performance; and physical functioning, relating to the whole range of daily and sporting activities linked to lifestyle [34]. Successful functioning in these areas makes it possible to activate the right coping strategies in the different contexts—family, school, and relationships—in order to better cope with the disease.

Coping Mechanisms. Children develop different strategies to manage their illness, typically classified as problem-focused coping, which involves actively addressing the source of stress (e.g., planning and seeking information), and emotion-focused coping, which aims to regulate emotional responses (e.g., distraction, avoidance, and seeking social support). In patients who see their illness as severe, perceiving themselves as vulnerable with increased anxiety and stressful behavior, improving psychological functioning and adaptive coping can lead to improved health status. The stress of living with a chronic disease could result, for example, in an increase in cortisol levels that affect physiological and behavioral development, and exacerbate some clinical conditions such as asthma or diabetes [35]. Behavioral problems may therefore develop, such as low self-esteem, anxiety, and depression, or other conditions directly linked to the diseases or associated with disease management [36].

Social and Academic Functioning. Poor socialization and poor school performance characterize children with chronic disease, due to a loss of motivation, increased school absences, and thus, lower academic achievement [37]. At school, some children turn out to be victims of bullying, prejudice, and stigmatization from peers, and these feelings lead, in many cases, to dropping out of school [38]. In cases where the chronic disease is affecting the nervous system, with direct effects on executive functioning skills, problems with attention, memory, and understanding cognitive processes are added to psychological impairments, resulting in exacerbation in medical adherence, therapies, and compliance with other recommendations [39].

Family and Sibling Impact. It is also necessary to observe these adverse psychosocial effects from the perspective of the family, in which a vicious circle is created, in which maladaptive dynamics develop alongside the protection that only the family unit can offer a child, both for the child with the disease and for the other members. For example, cognitive consequences and social isolation have been shown in parents [40]. In the case of congenital heart disease, several studies documented more pronounced psychological effects at the time of diagnosis, compared to parents of children with other diseases [41,42]. Of interest is that learning the diagnosis in the prenatal period was correlated with a higher maternal quality of life than receiving the diagnosis after the child’s birth [43]. In addition to the problems associated with the disease, there may also be several additional family factors which can further alter the condition of both the caregivers and the children themselves. In particular, economic problems, family conflicts, the need to manage a regular job, and the likelihood of reducing working hours or stopping work altogether all combine to create a condition that makes it difficult even to care for a child [44,45]. To these are also added effects on siblings. Some evidence reports that emotional difficulties, behavioral problems, hyperactivity, and inattention are more typical of healthy siblings of children with sensory impairment and not affected by other diseases, considering as a predictive factor the relationship between mother and sick child; the stronger this relationship, the greater the risk of the healthy sibling developing behavioral problems [46,47,48].

Thus, it is important to highlight how, in general, a negative disease perception, low self-esteem, and the lack of ability to manage the disease from patients and social context (family) increase the risk of developing comorbidities both during childhood and over the life span of a child, thus affecting cognitive functions, social development, relationships, and emotional regulation.

## 4. Coping with Chronic Disease in Daily Functioning

Children with chronic diseases experience some obvious limitations in managing normal activities due to recurrent pain, irregular development, medical treatment, and in some cases, frequent hospitalization, with severe consequences also in school achievement and peer activities [21]. These impairments are a consequence of the chronic nature of the disease, as described by Van Cleave et al.: “any physical, emotional, or mental condition that prevented him or her from attending school regularly, doing regular school work, or doing usual childhood activities or that required frequent attention or treatment from a doctor or other health professional, regular use of any medication, or use of special equipment” [49]. The impact of the disease on everyday life is strictly interconnected with the different phases of the chronic disease (diagnosis, treatment, and relapse). In fact, each period represents a challenge, but chronicity, potentially exerting greater psychological and physical stress than acute disease, needs better management [50,51]. Chronic disease is considered, according to the general model, as an allostatic load, characterized by physical and psychological wear and tear, but also by coping ability with the condition in order to prevent allostatic overload. In this section, we focus on the psychosocial and contextual risk factors such as maladaptive coping, school disengagement, parental distress, and economic strain that were most consistently identified in the literature as relevant to disease adaptation across multiple chronic conditions. These factors were selected based on their modifiability, clinical impact, and empirical recurrence in pediatric psychosocial research.

Whereas coping strategies in chronic diseases have been well studied in adulthood, less evidence has been found with respect to pediatric patients [52]. With respect to disease coping, it is important for the child to learn strategies to manage emotions and any behavioral problems associated with the disease, working on the controllability dimension [21]. For example, consider the case of diabetes, a disease that affects 1 in every 400 children, with recent evidence that suggests that the incidence is rising [53]. The guidelines recommend frequent monitoring of blood glucose levels, frequent insulin injections, monitoring of carbohydrate intake, and other lifestyle changes. Eating, for example, in the case of a diabetic child, involves a lot of attention. It is not possible to eat everything; the diet must be controlled and balanced. However, at that age, social gatherings are often centered around meals (e.g., family dinners and lunches with friends), recalling the illness and its limitations even more. This leads not only to increased stress levels that become chronic due to constant management and monitoring, but also to fear of the uncontrollable aspects of diabetes [54]. As a consequence, diabetic children feel different from their peers and end up feeling guilty [55]. Therefore, it is necessary to activate coping strategies both to control the disease and to learn how to deal with negative emotions related to the disease. One of the most studied strategies for children with chronic physical illness and related psychological comorbidities is cognitive behavioral therapy (CBT). This approach uses an active, directive, and structured methodology in order to teach little patients to become aware of their maladaptive cognitions and begin concentrating on real events linked to disease [56]. CBT interventions should be aimed precisely at reducing anxiety and stress, and the fear of being sick, of perceiving physical symptoms, to increase both treatment adherence and improve quality of life. Children with chronic diseases have matured their normal growth processes by developing maladaptive responses that can lead to altered behavior. It is important that they restore activities that increase self-confidence, facilitating cognitive change to reset their development. In the last years, among different types of coping strategies in the pediatric population, a growing body of research has focused on the possible role of spiritual coping, understood as the intimate and personal expression of the sacred, involving spiritual belief, behavior, and attitude to problem solve and manage stressful events [31]. While in the adult population, the data are very clear, suggesting how a spiritual approach can reduce physical health outcomes due to disease, in the pediatric population, the results are mixed [32]. Despite this discrepancy, mainly related to the fact that children have not yet developed critical thinking in this respect, spiritual coping is certainly a predictor of emotional and behavioral functioning, and thus may play an important role in the psychological and health functioning of pediatric populations with chronic illness [57]. Moreover, considering that parents also report negative emotional effects of dealing with their child’s illness, persisting in the long term for approximately 40% of subjects, it is also necessary for them to receive psychosocial support. A holistic approach should aim at improving coping and enhancing parenting. During routine medical examinations, physicians should also ask about the parents’ stress status, family functioning, and the child’s psychosocial functioning. When necessary, they should provide appropriate psychosocial assistance, such as educational interventions, cognitive behavioral techniques, and the strengthening of protective factors [41].

## 5. A Comprehensive Model for Health Management in Children with Chronic Disease

**Figure 1:**
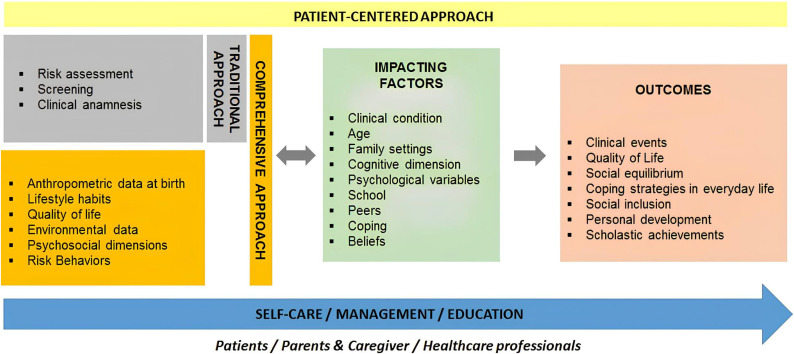
A widespread framework for health management in children with chronic disease.

Considering the individual variability in health behaviors and psychosocial outcomes of chronic disease management, it is pivotal to include the young patients in preventive and therapeutic health promotion actions, with the aim of mitigating or preventing the negative effects linked to the chronicity of the disease over time. Promoting self-management in children with chronic diseases represents a challenge for health care. Self-management is a multidimensional health intervention with a wide range of promotion activities. According to this approach, it is important that actions involve patients in the different contexts in which they relate, as if they were dyads or triads, i.e., within the family, the community, and the health system [58]. Acquiring the ability to self-manage diseases improves health behaviors, reduces the use of health services, and, as a result, improves the patient’s quality of life. However, although scientific evidence demonstrates positive effects of the self-management of chronic diseases in the pediatric population, there is little clarity and uniformity of methods. This is due especially to the precarious equilibrium in this population, not only because of the disease, but also because of the stage of development, changing social relationships, evolving cognitive functions, and hence the inability to activate appropriate coping strategies [59]. To achieve this, standardized tools and protocols are essential. These tools help assess the self-management skills of children and caregivers, identify environmental influences, and evaluate psychosocial and cognitive readiness. Standardization serves as a foundation—not a rigid framework—for personalized care. It reduces the risk of oversight or bias, ensuring that important aspects such as family dynamics, school participation, and emotional resilience are not neglected. While some may view standardization as overly prescriptive, it is best seen as a flexible starting point from which tailored interventions can be built. In clinical practice, comprehensive health management should include a risk assessment (e.g., psychosocial vulnerability and adherence barriers), family environment evaluation (e.g., conflict, support, and resources), school integration strategies (e.g., individualized education plans), coping and resilience training, and digital tools for self-monitoring and education (Figure 1). An example of such an integrated framework is the PENSAMI project, developed by the authors’ research group. This web-based tool focuses on the secondary prevention of pediatric patients with chronic diseases in order to more accurately stratify patients with a higher probability of events and to improve the quality of life of pediatric patients through a multidimensional health framework, including clinical, lifestyle, psychosocial, and environmental determinants [60]. The score adds to traditional clinical parameters, environmental factors, lifestyle habits, social context, emotional state, anthropometric measures at birth as preclinical signs to predict re-hospitalization, severity and progression of disease, quality of life, obesity and metabolic syndrome, school drop-out, and school performance. From the public health point of view, the development and implementation of an integrative approach represents a real step forward in individual awareness and empowerment in health promotion, starting from the patient’s own self-management to the awareness of health professionals and passing through the education of family members. The more a patient is involved in self-care behaviors, adopting effective behaviors and personalized and targeted coping strategies, the more favorable the results will be in terms of disease control and improved quality of life, both one’s own and that of the family (Figure 1).

## 6. Limitations and Future Perspectives

In this narrative review, we outlined some points regarding the definition and burden of disease in pediatric populations, the experience of chronic disease, and the implications from a psychosocial point of view, both for patients and for families (Table 1). However, this review was not conducted with a systematic approach, and this may be a limitation in the conclusion context. It is important to highlight the need, even in the pediatric population, to develop a patient-centered approach, thus taking into account not only the clinical dimension but the human dimension in general (the perception of well-being and the impact of the disease on daily life, quality of life, and social context). It is also pivotal to emphasize that children with a chronic disease experience their condition in an already delicate developmental window and are therefore at greater risk of developing one or more adverse effects related to the disease, treatment, or maladaptive health behavior, both in the short term but especially in the long term. Poor management of the disease during childhood, on the part of the patient, but especially on the part of the social and emotional context in which the patient lives, will increase the risk of developing adverse effects.

## 7. Conclusions

Children diagnosed with chronic illnesses experience their condition during a critical developmental window, making them vulnerable to emotional, cognitive, and social disruptions. Effective management must go beyond clinical indicators, integrating education, self-management, and psychosocial support into a holistic framework.

A patient-centered, multidimensional approach—one that recognizes the interconnected roles of clinical, familial, educational, and social environments—is essential. Only by addressing these factors together can we improve not just survival, but the quality of life and long-term well-being of children and families affected by chronic illness. Further studies are recommended to contextualize, develop, and validate these models, exploring the real effects on functioning and daily experiences.

## Figures and Tables

**Table 1:** General results of the main studies included.

Main Author	Type of Article	Study Population	Materials and Methods	Main Results
Wagner [4]	Research Article	Children with CD	In a representative sample of Austrian adolescents aged 10–18 years, internalizing, externalizing, and behavioral problems were assessed cross-sectionally using the Youth Self-Report and health-related quality of life (HrQoL) using the KIDSCREEN questionnaire.	Of 3469 adolescents, 9.4% of girls and 7.1% of boys suffered from a chronic pediatric illness. Of these individuals, 31.7% and 11.9% had clinically relevant levels of internalizing and externalizing mental health problems, respectively, compared to 16.3% and 7.1% healthy adolescents. Anxiety, depression, and social problems were twice as high in this population.
Quintana [48]	Systematic Review	Siblings	/	The siblings of those with chronic illnesses have higher reported emotional, behavioral, and social problems than those with healthy siblings.
Queen Mary University of London [8]	Research Article	Children with CD	The results involved 7000 children. The measure of chronic illness was based on mothers assessing their child’s health at 10 and 13.	Children with CD were approximately twice as likely at 10 and at 13 to present with a mental health disorder than the control group (children reported by their mothers to be ‘healthy, no problems’). At age 15, children with chronic health problems were 60% more likely to present with such disorders.
Cohbam [36]	Systematic Review	Children with CD	/	Given the burden of disease of anxiety disorders, regardless of the impact on the disease outcomes, screening for and treatment of anxiety is recommended in youths with chronic medical conditions.
Caliendo [47]	Research Article	Parents	The results involve 153 children from the region of Campania and their caregivers through the administration of the Strength and Difficulties Questionnaire.	From the data, it emerged that siblings of children with autism spectrum disorder and siblings of children with Down syndrome have a greater emotional fragility, especially among male subjects.
Shojaee and Alizadeh [46]	Research Article	Siblings	The sample included all siblings of children with and without sensory impairment (SI). The sample consisted of 91 subjects: 38 siblings of children with SI and 53 without SI.	Siblings of children with SI are significantly at a higher risk of psychological diseases and need support and services.
Fayed [11]	Research Article	Children with CD	QUALITÉ cohort study includes 6 Canadian child epilepsy ambulatory programs with a total sample of 3481 children.	QOL is strongly related to mental health and social support in children with CD, but not to their seizures.
Jackson [42]	Systematic Review	Parents	/	A holistic approach should aim at improving adaptive capacities and productive parenting practices. This should lay a solid basis for these families to successfully deal with future challenges and uncertainties at various stages in the trajectory of the child’s status.
Denny [23]	Research Article	Children with CD	A sample of 9107 students (Years 9–13) from 96 New Zealand high schools. Students were asked about any chronic illnesses or disabilities.	One in five students reported having a chronic health condition; of these, 28% reported a negative impact of the disease on their daily activities, and 8% reported an impact on their ability to socialize.
Hajek [39]	Research Article	Children with CD	Participants included 36 children with perinatal or childhood arterial ischemic stroke and a comparison group of 15 children with asthma. Outcomes included cognitive ability, executive functions, and neurological function (Pediatric Stroke Outcome Measure). Magnetic resonance imaging measured lesion location and volume.	Following arterial ischemic stroke, children performed at the low end of the average range on measures of cognitive functioning. Cognitive outcomes depend on a variety of factors.
Spencer [45]	A cohort study	Parents	The study commenced in 2004 with two cohorts: families with 0–1-year-old infants (the B cohort) and families with 4–5-year-old children (the K cohort). Interviews took place in the family home with the main respondent, usually the mother (99%).	This study is consistent with published literature in that it finds there is an impact on parental work status of caring for a child with chronic health problems in the early years of life. The work status of both parents was adversely affected, although in different ways.
Kelo [9]	Research Article	Children with CD	Education protocols for school-age children with diabetes by nurses.	The education of patient management process was successfully described.
Forrest [37]	Research Article	Children with CD	A total of 1457 children in the fourth through sixth grades from 34 schools in 3 school districts and their parents provided survey data; parents completed the Children With Special Health Care Needs Screener.	In total, 33% of children screened positive for special health care needs. They experienced significantly lower academic achievement, as measured by grades, standardized testing, and parental-assessed academic performance.
Van Cleave [50]	A cohort study	Children with CD	Cohort is represented by National Longitudinal Survey of Youth-Child Cohort (1988–2006). Children were aged 2 through 8 years at the beginning of each study period, and cohorts were followed up for 6 years.	Prevalence of CD increased from 1988 to 2006. However, presence of these conditions was dynamic over each 6-year cohort.
Marin [51]	Research Article	Children with CD	A total of 71 children with asthma and 76 medically healthy children completed interviews regarding life stress, and peripheral blood samples were collected. After mononuclear cells had been mitogenically stimulated, production of the cytokines IL-4, IL-5, IL-13, and IFN-γ was measured. All measurements were repeated every 6 months for two years.	Children with asthma who had higher levels of chronic family stress showed increased production of IL-4, IL-5, and IFN-γ at times when they had experienced an acute event compared to times when they had not. These stress-related changes did not occur in asthmatic children with lower levels of chronic family stress or in healthy controls.
Harjutsalo [55]	A cohort study	Children with CD	Cohort study is represented by children with type 1 diabetes listed on the National Public Health Institute diabetes register, Central Drug Register, and Hospital Discharge Register in 1980–2005.	The incidence of type 1 diabetes is increasing even faster than before. The number of new cases diagnosed at or before 14 years of age will double in the next 15 years, and the age of onset will be younger (0–4 years).
Varni [14]	Research Article	Children with CD	The analyses were based on over 2500 pediatric patients from 10 physician-diagnosed disease clusters.	Pediatric patients with CD reported progressively more impaired overall HRQOL than healthy children with medium-to-large effect sizes.
Van Cleave and Davis [38]	Research Article	Children with CD	A secondary data analysis using the National Survey of Children’s Health of >102,000 US households, measuring the association between having a special health care need and being a victim of bullying.	Overall, 21% of children with CD reported being bullied, resulting in chronic behavioral, emotional, or developmental problems.
Lawoko and Soares [41]	Prospective longitudinal study	Parents	/	A significant proportion of parents of children with congenital heart disease are at risk of long-term psychosocial morbidity, with a need to implement psychosocial interventions.

## Data Availability

Not applicable.
